# Alarm from Rumor of the Prevalence of Child-Bed Fever

**Published:** 1873-04

**Authors:** 


Alarm from Rumor of the Prevalence of Child-Bed Fever.—We copy from the "Courier" the following correspondence, which explains itself, and shows how that even the false rumor of a pestilence may produce as great a panic as pestilence itself:

Buffalo, April 25, 1873.

My Dear Sir—From several sources, professional and otherwise, I hear that you have been very unfortunate recently in cases of puerperal fever, and that you contemplate abandoning this class of practice. If you think it proper to notice this prevalent rumor I shall be glad to make your reply public.

Sincerely yours,

Joseph Warren.

Thomas F. Rochester, M. D.

Dr. Rochester's reply.

April 25th, 1873.

My Dear Mr. Warren—Your favor of this date is received. Inasmuch as a few malicious and evil-disposed individuals, and a great many thoughtless and gossiping persons, have most industriously circulated reports, to the great alarm and detriment of my patients, as also to my own personal prejudice to a very limited extent, to the effect that I have been very "unfortunate" and have lost a large number of cases of child-bed fever, (variously stated at from eight to twenty a week) I am constrained for the first time in my life to appear in a public print, as affording the only channel to present the truth of the matter and to deny and refute a slander injurious to the public welfare and reflecting on me professionally. This I do, by the simple and unqualified statement, that within the past year, up to this moment, I have lost but two lying-in patients one by child-bed fever and one (who was convalescing from that disease) by shock, from the sudden death of her infant with measles, and by the non-elimination of the measles poison from her own system, she never having had that disease, and presenting, during the last twenty-four hours of her life, that dullness and stupor characteristic of measles, when, as the popular phrase is, "it does not come out." Both of these patients died more than a month ago, and when the second one was confined, the first one presented no systoms whatever of child-bed fever. During the illness of these patients, and for twenty days subsequent to the death of second, I positively refused to attend any persons in confinement, being determined to avoid any possibility of conveying the disease, acting as I have always done and advocated for twenty-five years, and obeying a cardinal law of every well-informed and conscientious physician. Since then I have attended several, all of whom are doing well and have presented no indications of child-bed fever. In reply to one of your interrogatories, I answer, I do not " contemplate abandoning this class of practice." Those persons who *desire* my services (and I wish for none others as patients) can have them as heretofore, and need not fear that I will convey to them disease or be more "unfortunate" than others in the treatment of it when developed from atmosphiric or other sources. I must again refer to publishing in a newspaper what, to a certain extent, is a personal matter; but I see no other method of meeting this most outrageous and wide-spread slander. Did I know surely with whom it originated, I would take another course with regard to it. Yours, respectfully,

THOMAS F. ROCHESTER.

